# Intercropping of Peanut–Tea Enhances Soil Enzymatic Activity and Soil Nutrient Status at Different Soil Profiles in Subtropical Southern China

**DOI:** 10.3390/plants10050881

**Published:** 2021-04-27

**Authors:** Taimoor Hassan Farooq, Uttam Kumar, Jing Mo, Awais Shakoor, Jun Wang, Muhammad Haroon U. Rashid, Muhammad Aammar Tufail, Xiaoyong Chen, Wende Yan

**Affiliations:** 1 National Engineering Laboratory for Applied Technology of Forestry and Ecology in South China, Central South University of Forestry and Technology, Changsha 410004, China; taimoorhassan2055@gmail.com (T.H.F.); jingmo_changsha@hotmail.com (J.M.); jwang0829@csuft.edu.cn (J.W.); 2 College of Life Science and Technology, Central South University of Forestry and Technology, Changsha 410004, China; 3 Institute of Applied Ecology, College of Plant Protection, Fujian Agriculture and Forestry University, Fuzhou 350002, China; Uttam5454@gmail.com; 4 Department of Environment and Soil Sciences, University of Lleida, Avinguda Alcalde Rovira Roure 191, 25198 Lleida, Spain; awais.shakoor@udl.cat; 5 College of Forestry, Fujian Agriculture and Forestry University, Fuzhou 350002, China; haroonrashid3838@outlook.com; 6 Department of Civil, Environmental and Mechanical Engineering, University of Trento, via Mesiano 77, 38123 Trento, Italy; muhammad.tufail@unitn.it; 7 College of Arts and Sciences, Governors State University, University Park, IL 60484, USA

**Keywords:** *Camellia oleifera*, *Arachis hypogaea*, soil nutritional status, soil quality, cropping pattern, silvicultural methods, sustainable production

## Abstract

Intercropping is one of the most widely used agroforestry techniques, reducing the harmful impacts of external inputs such as fertilizers. It also controls soil erosion, increases soil nutrients availability, and reduces weed growth. In this study, the intercropping of peanut (*Arachishypogaea* L.) was done with tea plants (*Camellia oleifera*), and it was compared with the mono-cropping of tea and peanut. Soil health and fertility were examined by analyzing the variability in soil enzymatic activity and soil nutrients availability at different soil depths (0–10 cm, 10–20 cm, 20–30 cm, and 30–40 cm). Results showed that the peanut–tea intercropping considerably impacted the soil organic carbon (SOC), soil nutrient availability, and soil enzymatic responses at different soil depths. The activity of protease, sucrase, and acid phosphatase was higher in intercropping, while the activity of urease and catalase was higher in peanut monoculture. In intercropping, total phosphorus (TP) was 14.2%, 34.2%, 77.7%, 61.9%; total potassium (TK) was 13.4%, 20%, 27.4%, 20%; available phosphorus (AP) was 52.9%, 26.56%, 61.1%; 146.15% and available potassium (AK) was 11.1%, 43.06%, 46.79% higher than the mono-cropping of tea in respective soil layers. Additionally, available nitrogen (AN) was 51.78%, 5.92%, and 15.32% lower in the 10–20 cm, 20–30 cm, and 30–40 cm layers of the intercropping system than in the mono-cropping system of peanut. Moreover, the soil enzymatic activity was significantly correlated with SOC and total nitrogen (TN) content across all soil depths and cropping systems. The depth and path analysis effect revealed that SOC directly affected sucrase, protease, urease, and catalase enzymes in an intercropping system. It was concluded that an increase in the soil enzymatic activity in the intercropping pattern improved the reaction rate at which organic matter decomposed and released nutrients into the soil environment. Enzyme activity in the decomposition process plays a vital role in forest soil morphology and function. For efficient land use in the cropping system, it is necessary to develop coherent agroforestry practices. The results in this study revealed that intercropping certainly enhance soil nutrients status and positively impacts soil conservation.

## 1. Introduction

The enhancement and maintenance of soil productivity and sustainability through the long-term use of different silvicultural practices, such as varying planting density and spacing, introducing native and exotic beneficial species, agroforestry and intercropping, has been widely used worldwide [1,2,3,4,5,6]. Intercropping can enhance soil quality by incorporating a significant amount of topsoil and subsoil organic matter and releasing and recycling nutrients [7,8,9]. It is the general opinion that intercropping is more suitable than mono-cropping for the long-term maintenance of soil fertility [10]. Intercropping systems are rarely dependent on external inputs such as fertilizers [11]; this also reduces the adverse environmental effects such as soil erosion [12]. In China, trees/legume intercropping is most commonly used because it reduces crop failure risk and improves land use [13].

Tea (*Camellia oleifera*) is an evergreen shrub of the Theaceae family, and it is widely found in central and south China [14]. Moreover, it can also survive in nutrient-depleted soils. Peanut (*Arachishypogaea* L.) is a leguminous crop that can fix the atmospheric N and increase soil fertility [15]. According to the US Department of Agriculture, it produces a considerable amount of organic N, improves soil organic matter (SOM), helps in nutrient release and recycling, and improves soil structure. When peanut was intercropped with maize, it played a crucial role in changing soil health by influencing soil microbes composition [16]. Additionally, the dominant microbial species altered due to the peanut intercropping, which shows a close and significant relationship between improving available soil nutrients and soil enzyme activities [17].

Soil enzymes are continually playing dynamic roles in the maintenance of soil health. Soil enzymes are the direct mediators for the biological catabolism of soil organic and mineral components. They are often closely associated with SOM, soil physical properties, and microbial activities and biomass. They are the better indicators of soil health as changes in enzymes occurred much earlier than other soil parameters, thus providing early indications of changes in soil health. Their activities can also be used as measures of microbial activity and soil productivity [18,19]. Although they are present in a very nominal quantity, their role in soil quality can never be ignored. Likewise, a soil nutrient’s total content and soil nutrient availability directly affect the plant’s growth and development, reflecting soil health [20,21,22]. In the southern Chinese province of Hunan, the adoption of agroforestry (intercropping) in tea interests the growers because intercropping controls weed growth and soil erosion. However, many of the sites where these plantations were established are deficient in available macronutrients, albeit a high level of total phosphorus (P). The available P rapidly forms insoluble complexes with cations, particularly aluminum and iron. Less nutrient availability in forest soils is considered as one of the most important causes of productivity decline [23]. Nutrient total status and availability are essential for plant growth and development, and thus, macronutrient availability is vital for sustainable productivity. Soil enzymes play a significant role in nutrient recycling in soil ecosystems. In addition, soil enzymes play a crucial role in maintaining soil quality. They can provide essential and early detection signals for soil metabolic activity and nutrient status changes. However, few publications have focused on how legume intercropping with tea plantation, specifically related soil enzymatic activities, influences soil fertility.

Therefore, the objectives of our current study were (1) to investigate the soil total nutrient content and soil nutrient availability; (2) and the activities of soil enzymes (sucrase, protease, urease, acid phosphatase, and catalase); and (3) to explore a correlation between soil enzymatic activity and soil nutrients in different cropping systems and soil depths. Three cropping systems, including peanut–tea intercropping, tea mono-cropping, peanut mono-cropping, and four different soil depths (0–10 cm, 10–20 cm, 20–30 cm, and 30–40 cm) were used in this study. Our hypotheses were that: (i) intercropping favors better nutrient availability and total nutrient content than monoculture systems due to better litter quality and decomposition, and nutrient availability will decrease with the increase in soil depth; (ii) soil enzymatic activity will be higher in the peanut–tea intercropping system than in tea monoculture because peanut influences soil microbial composition—thus, it can enhance soil microbial activity, which has a significant relationship with the improvement of soil enzyme activities; and (iii) there will be a positive and considerable relationship between soil nutrients and soil enzymatic activity across all cropping types and soil depths.

## 2. Results

### 2.1. Soil Organic Carbon

Among all the cropping systems and soil depth, SOC content ranged from 5.61 g.kg^−1^ to 19.93 g.kg^−1^. The topmost layer had the highest SOC content among all the cropping systems. While comparing each cropping system at the same depth, the SOC content was significantly different except at a 10–20 cm depth of CPM and CMM. SOC content decreased as the soil depth increased in the PMM and CMM. However, in CPM, it was observed to be highest at 20–30 cm. Overall, SOC was with the order of PMM = CPM > CMM (Table 1).

### 2.2. Fluctuations in Soil Total Nutrient Status

TN ranged from 0.72 g.kg^−1^ to 1.51 g.kg^−1^ among all cropping types and soil depths. PMM had the highest TN content among all the cropping systems at the topmost layer. TN content decreased as the soil depth increase, apart from CPM, where it was observed as the highest at 20–30 cm. There was a significant difference present in TN at each soil layer among the three cropping systems, except at a 10–20 cm depth of CPM and CMM. In terms of TP, among all the cropping patterns and soil depths, its content ranged from 0.21 g.kg^−1^ to 0.57 g.kg^−1^. CPM had the highest TP observed at 10–20 cm.

**Figure 1:**
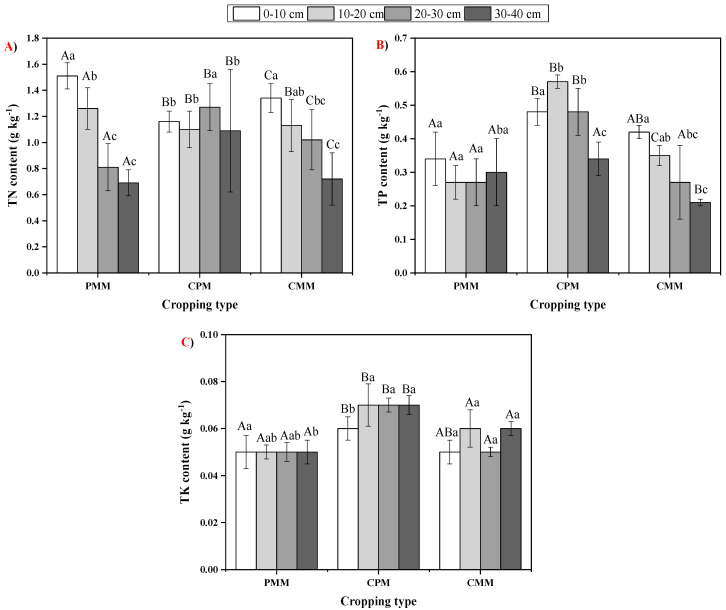
Variability in (**A**) soil total nitrogen (TN), (**B**) total phosphorus (TP) and (**C**) total potassium (TK) content at different soil depths in *C. oleifera* mono-cropping model (CMM), peanut mono-cropping model (PMM), and Camellia–peanut inter-cropping model (CPM). Two-way analysis of variance (ANOVA) test was conducted and the values in the columns are mean ± SD. Different uppercase letters indicate significant differences between the cropping systems at *p* < 0.05, while the different lowercase letters indicate significant differences among the soil depths *p* < 0.05.

The TK content ranged from 0.05 g.kg^−1^ to 0.07 g.kg^−1^ among all soil layers and cropping patterns. CPM had the highest TK content, and it was observed to be the highest at 10–20 cm depth. At the same depths, there was a non-significant difference observed between PMM and CMM; however, both were significantly different to PMM. Overall, the TP and TK contents were in the order of CPM > CMM > PMM (Figure 1).

### 2.3. Variability in Soil Nutrient Availability

**Figure 2:**
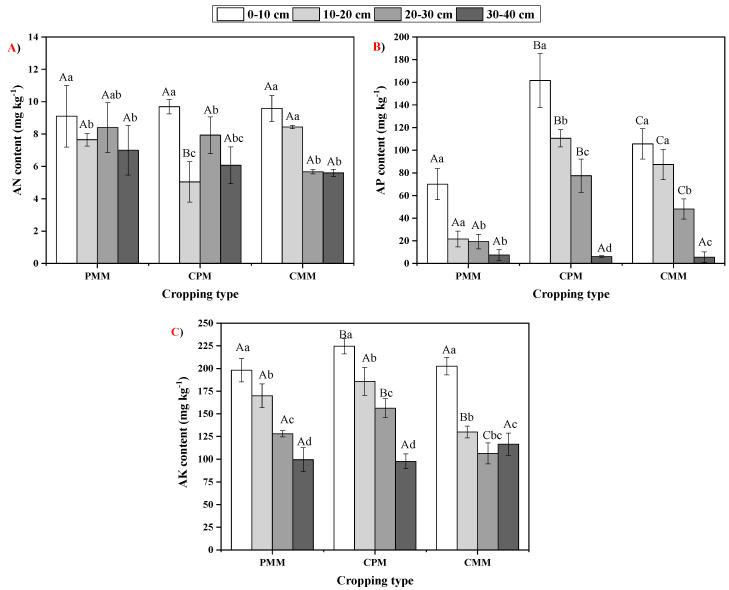
Variability in (**A**) soil available nitrogen (AN), (**B**) available phosphorus (AP) and (**C**) available potassium (AK) content in different soil depths in *C. oleifera* mono-cropping model (CMM), peanut mono-cropping model (PMM), and Camellia–peanut inter-cropping model (CPM). Two-way analysis of variance (ANOVA) test was conducted and the values in the columns are mean ± SD. Different uppercase letters indicate significant differences between cropping systems at *p* < 0.05, while different lowercase letters indicate significant differences among soil depths *p* < 0.05.

Among all the soil layers and cropping patterns, AN content ranged from 5.04 mg.kg^−1^ to 9.69 mg.kg^−1^. There was a non-significant difference observed for AN between different cropping systems at each soil layer, except 10–20 cm depth. The AP content ranged from 7.55 mg.kg^−1^ to 110.58 mg.kg^−1^ among all the soil depths and cropping systems, and it was observed to be the highest in the topsoil layer. Overall, the AP content was observed highest in CPM. Similar to AP, AK was also observed to be higher in CPM compared to both mono-cropping systems. On average, the AN content followed the order of PMM > CPM > CMM, whereas AP content followed the order of CPM > CMM > PMM, and the AK content followed the order CPM > PMM > CMM (Figure 2).

### 2.4. Soil Enzymatic Responses in Different Cropping Types

**Figure 3:**
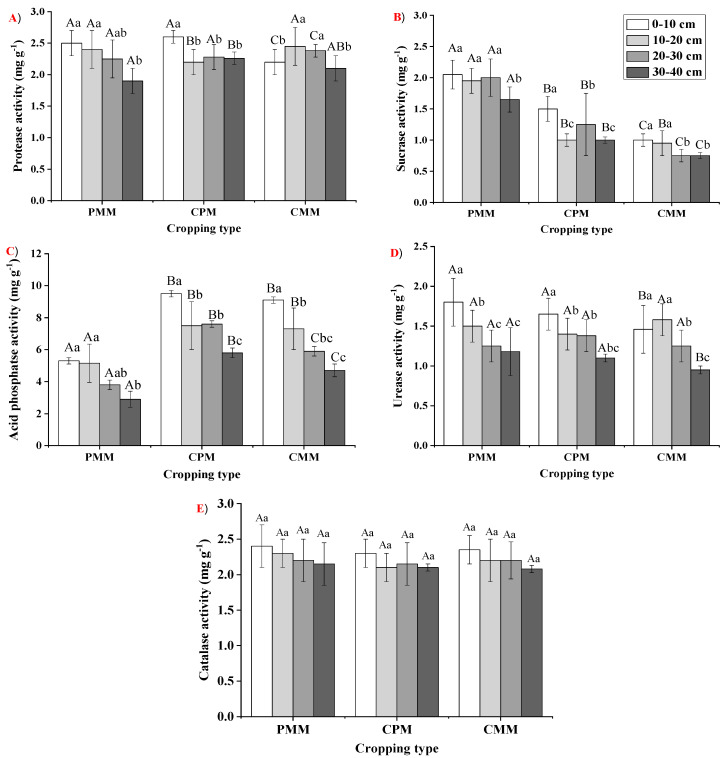
The activity of soil enzymes (**A**) protease; (**B**) sucrase; (**C**) acid phosphatase; (**D**) urease; and (**E**) catalase (mg.g^−1^) at different soil depths in the three cropping models in the study site. A two-way analysis of variance (ANOVA) test was conducted and the values in the columns were the mean ± SD. Different uppercase letters indicated significant differences between the cropping systems at *p* < 0.05, while different lowercase letters indicated significant differences among soil depths *p* < 0.05.

All enzymes had the highest activity in 0–10 cm for each cropping system except CMM. In CMM, soil urease and protease activity were most increased at a 10–20 cm depth. The protease content varied significantly for all the cropping systems in the 0–10 cm and 30–40 cm layer and was observed to be the highest in CPM. The sucrase content followed the order PMM > CPM > CMM in all soil layers. For acid phosphatase, all layers followed the order CPM > CMM > PMM. The urease activity varied significantly among all cropping systems in the 0–10 cm, 10–20 cm, and 30–40 cm layers; only in the 20–30 cm layer was there no significant difference observed. Catalase content was not significantly different in the 0–10 cm, 20–30 cm and 30–40 cm among the cropping systems, and it was the highest in PMM. However, in the 10–20 cm layer, it was observed highest in CPM (Figure 3).

### 2.5. Correlation between Soil Nutrients and Soil Enzymatic Activity

(1) In PMM, sucrase, protease and catalase had no significant correlation with any nutrient. Urease was positively correlated to SOC, TN and AK, while negatively correlated to TK. Acid phosphatase was positively correlated with SOC, TN and AK. (2) In CPM, sucrase was positively related to TN; protease to SOC; urease to SOC, AN and AK; acid phosphatase to SOC; and catalase to SOC and AK. (3) In CMM, sucrase was positively related to AP, while protease was negatively associated with AN. Urease, acid phosphatase and catalase enzymes were positively associated with SOC, TN, TP, AP and AK (Table 2).

### 2.6. Path Coefficients between Soil Nutrients and Soil Enzyme Activity

In PMM, among all the soil nutrients, the direct path coefficients of soil TN (0.821) and SOC (−0.534) on soil sucrase activity were relatively large. The direct path coefficients of SOC (0.689) and AP (0.524) indicate the substantial direct effect of these nutrients on protease activity. SOC also has a robust direct path coefficient with acid phosphatase activity (1.045). The direct path coefficients of SOC (2.203) and TN (−2.069) on catalase activity indicate that both had strong positive and negative effects on catalase activity, respectively (Table A1).

In CPM, soil TN, SOC and AN had a robust positive effect on sucrase enzyme activity. However, the path coefficient was small. SOC (0.469) had a sizeable direct path coefficient with protease indicates the substantial direct effect on protease activity. Moreover, SOC (0.7074) also has a robust positive path coefficient with urease activity. The direct path coefficients of all soil nutrients with catalase activity were also positive. Compared with the other four enzymes, the direct and indirect path coefficients of soil nutrient factors with acid phosphatase were relatively small, indicating that soil nutrient factors had a more negligible effect on acid phosphatase activity (Table A2).

In CMM, soil AP (0.532) had a more significant direct path coefficient on sucrase and TP (0.905), which strongly affected protease activity shows. The effect of soil nutrients on urease activity was positive with a relatively more minor path coefficient. The direct path coefficient of AP (0.885) on acid phosphatase was rather significant. In addition, TN (0.436) had a direct effect on catalase activity (Table A3). Two-way ANOVA results of the testes soil variables are shown in Table 3.

## 3. Discussion

In the present study, SOC was unanimously higher in the 0–10 cm soil layer among all the cropping systems because of the higher litterfall on the topmost layer. Compared to the mono-cropping of tea, SOC was higher in peanut–tea intercropping, possibly due to the higher litterfall and tea biomass decomposition inputs. Growing different crops on the same land simultaneously helps maintain the SOC and improve nutrient cycling [24,25]. A similar kind of increase in tea soil SOC content was also observed by [26,27]. N is vital for plant growth and development [22,28]. It is available in many soil forms, such as nitrate and ammonia [29,30]. Peanut being a legume plant could fix atmospheric nitrogen [31,32] and as a result, we found out that PMM had the highest amount of TN for the upper two layers, while in the remaining two layers, it was observed to be highest in CPM. This could be due to the tea plant deep root system, which makes way in the soil deeper layers for the atmospheric N that the peanut plant fixes. Both TP and TK contents were higher in the CPM among all the soil layers than the tea and peanut mono-cropping system. The probable reason was that intercropping can provide a much more comprehensive ground cover along with better water use efficiency [31,32].

Leguminous plants can convert the unavailable N form into a useable form [33]. The comparison of CPM and PMM depicted that AN was 51.78%, 5.92%, and 15.32% lower in the 10–20 cm, 20–30 cm, and 30–40 cm layers of the intercropping system than in the peanut mono-cropping system because the of higher urease activity in CPM, which led to an increase in the NH_3_ loss from soil [34,35]. AP in the intercropping of tea and peanut was higher in all the soil layers than the tea and peanut monoculture cropping. This is because leguminous plant led to the acidification of the rhizosphere with the help of roots, and these roots release the organic acids [36,37,38,39]. Due to acidification, the enzyme acid phosphatase starts the dissolution of P-based minerals, which increases the P availability. Maurya and Lal [40] obtained similar results, where the amount of P increased due to the release of acid phosphatase by the chickpea, which led to the conversion of organic P into inorganic P. AK content was higher in CPM compared to CMM and PMM in all soil layers. AK was 11.1%, 43.06%, 46.79% higher in 0–10 cm, 10–20 cm, and 20–30 cm layers of the intercropping system than the mono-cropping of tea. Similarly, AK was 13.4%, 9.29%, and 22.06% higher in 0–10 cm, 10–20 cm, and 20–30 cm layers of intercropping system than the mono-cropping system of peanut. The increase in AK in the intercropping system can be attributed to the increase in the soil enzymatic activity.

Soil enzymes play a significant role in the soil ecosystem’s overall biochemical functioning [41,42,43]. A better understanding of the soil enzymatic activity in different cropping systems with a depth effect can provide better knowledge about how intercropping systems can improve soil fertility. It is evident from the previous studies that the mono-cropping system could potentially harm the soil enzyme mechanism, which results in a significant decrease in the soil enzymatic activity [44]. In our current study, a very considerable increase in the enzyme activities was observed in the intercropping system of the tea and peanut than in the mono-cropping systems. Protease is the main enzyme involved in the catalysis of N minerals and N cycling [45]. The protease activity was higher in the layers of 0–10 cm and 30–40 cm for the intercropping system than both mono-cropping systems. This change in protease activity might be attributed to a higher SOC content in the topsoil [46]. However, in the 30–40 cm layer, the increase might be due to a higher N content. Soil sucrase enzymes catalyze sucrase to glucose and fructose with hydrolysis, and it is also connected with the biomass of the soil microbes [47,48]. The mono-cropping of peanut had the highest sucrase enzyme activity, possibly due to a higher SOM. These findings are supported by Li et al. [41], who observed that soil enzyme activity was significantly enhanced due to intercropping. Acid phosphatase is the main enzyme involved in ester hydrolysis and phosphoric acid anhydrides [49,50]. It converts the esters and anhydrides into phosphate, and is a key enzyme in P cycling in soil [51].

In our current study, acid phosphatase activity was higher in the intercropping of peanut–tea than tea mono-cropping because the peanut was identified as a species whose roots release an ample amount of acid phosphatase in the soil [52,53]. The soil urease enzyme catalyzes urea into NH_3_ and CO_2_ with hydrolysis [54]. This is a necessary process that regulates N availability to the plants after the application of urea. To some level, urease indicates the availability of N in different cropping systems. The catalase’s primary function is to decompose the organic matter into the plant’s useable form [55,56]. In our current study, the mono-cropping of peanut had a higher activity of urease and catalase than intercropping because of the SOM content of the mono-cropping of peanut. In this study, the enzyme activity was more or less associated with the content and distribution of SOC, TN and AK among all the cropping systems. Our results are in line with Tian et al. [57] and Udawatta et al. [58]; they also observed a stronger correlation between SOC and enzymes. An increase in the SOM and litter quantity enhances the soil activity [59,60] and this increment has direct involvement in the improvement of nutrient cycling, along with greenhouse emission [61,62,63], which, in return, has a positive impact on the ecosystem, plant growth and overall SOC.

## 4. Materials and Methods

### 4.1. Study Site

**Figure 4:**
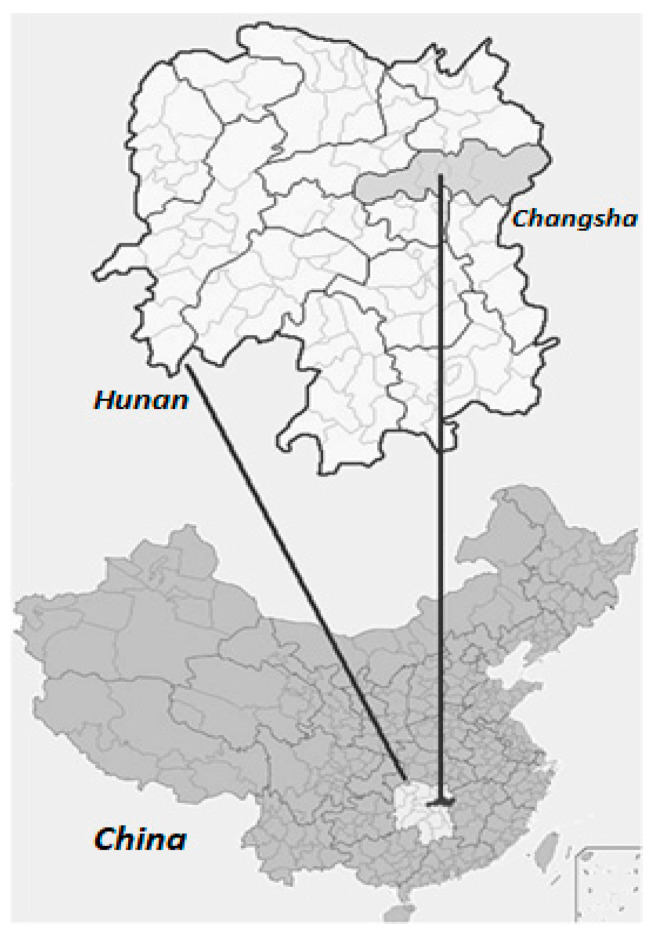
Location of the study area, Hunan botanical garden, Changsha, Hunan.

The study was conducted in Hunan Botanical Garden in Changsha city, Hunan Province, China (113°02′–113°03′ E, 28°06′–28°07′ N) (Figure 4). The study area had a typical subtropical humid monsoon climate, with a mean annual precipitation of 1378 mm, mean annual temperature of 17.2 °C, and a mean annual relative humidity of 81%. The mean annual average sunshine was 1814.8 h and the frost-free period was 275 days. The site was the hillside, with a slope of about 5–15°. The soil was classified as typical red earth developed from the quaternary red clay reticulated parent material. The soil texture ranged from clay loam to sandy loam, with a depth of about 1 m. The soil was acid with a pH of 4.5–5.5. Soil bulk density was ranged between 1.16 to 1.22 (g/m^3^).

### 4.2. Experimental Design

In this study, three cropping models were set up which included: (1) a *C. oleifera* mono-cropping model (CMM); (2) a peanut mono-cropping model (PMM), and (3) a Camellia–peanut inter-cropping model (CPM). The *C. oleifera* monoculture stand was established in 2010, and the line-row spacing of the *C. oleifera* was planted in 4 m × 3 m. In CMM, an area with three lines and three rows of *C. oleifera* trees was selected as a plot (about 110 m^2^). Four plots were set up for CMM in this study.

However, in PMM, the peanuts were planted in the open space beside the *C. oleifera* forests according to the 0.25 m × 0.1 m spacing in April 2018. An area with 40 lines and 100 rows of peanut plants was selected as a plot (about 100 m^2^). Four plots were set up for PMM in this study. Moreover, in CPM, an area with three lines and three rows of *C. oleifera* trees was selected as a plot (about 110 m^2^). Peanuts were intercropped between *C. oleifera* trees in April 2018. The spacing between the peanut and tea plant was 1 m. Four plots of CPM were set up in this study. The three cropping models employed similar field management practices during the study period.

### 4.3. Soil Sampling

Soil samples were collected in September 2019. Four plots of 20 × 20 m were established in each of CMM, PMM, and CPM plots; four pits were dug diagonally. A soil corer was used to obtain soil samples from 0–10, 10–20, 20–30, and 30–40 cm depths from each pit. Four replicates were taken from each plot for each depth. The rocks and plant residues were removed from the soil samples. Soil samples were placed into self-sealing plastic bags, labelled, and delivered to the laboratory for further analysis. For enzymatic activity analysis, the soil samples were placed in −80 °C until further use. For nutrients analysis, after air drying, the soil samples were passed through a 0.15 mm and 0.20 mm sieve for the determination of soil organic carbon (SOC), total nitrogen (TN), total phosphorus (TP), total potassium (TK), available nitrogen (AN), available phosphorus (AP), and available potassium (AK).

### 4.4. Soil Chemical Analysis

Urease activity was measured following the method described in [64]. Five grams of soil was incubated with 10 mL of citrate phosphate buffer (pH 6.7) and 5 mL of 10 % urea solution at 38 °C for 3 h. Activity was determined by measuring the released NH_4_^+^ with a spectrophotometer at 578 nm. Acid phosphatase activity was analyzed with nitrophenyl phosphate disodium (PhOH mg g^1^, 37 °C, 24 h), and catalase with KMnO_4_ (0.1 mol L^−1^ KMnO_4_ ug g^−1^, 30 °C, 20 h) [65]. Sucrase activity was determined by the method of [66]. For sucrose, the air-dried soil (5 g) was incubated with 15 mL sucrose. Five microliters (5 mL) of phosphate buffer (pH 5.5) and five drops of toluene at 37 °C for 24 h, and the reaction solution was filtered through the quantitative filter paper as rapidly as possible after incubation. Filtrate (1 mL) was mixed with 3 mL salicylic acid at 100 °C for 5 min in the water bath, and the mixture was adjusted to 50 mL and cooled with deionized water. Sucrase activity was determined spectrophotometrically at 508 nm. The protease activity was determined by ninhydrin colorimetry, expressed in milligrams of amino nitrogen in 1 g of soil cultured for 24 h in a 37 °C incubator [67].

SOC was determined by the hydrated potassium thermo-dichromate oxidation method. TN was determined using the CN elemental analyzer, while the colorimetric method of the molybdenum–antimony solution with royal acid was used to determine TP. The flame photometer method was used to determine TK. AN was analyzed by the Kjeldahl method [68]; AP was determined by the diacid extraction spectrophotometric colorimetry method [69], and AK was determined using a flame photometer method by ammonium acetate extraction [70]. Detailed information about the total and available nutrients was also mentioned in our published paper [6].

### 4.5. Statistical Analysis

Two-way analysis of variance (ANOVA) was performed to analyze the difference in soil enzymatic activity, soil total nutrient status and soil nutrient availability between different cropping systems at four different soil depths. Correlation analysis was performed using the Pearson statistical method to analyze the association among the studied soil parameters. Moreover, the path analysis using multiple linear analysis was also performed to study the relationships betweesn soil enzyme and soil nutrients. Results were declared statistically significant at *p* < 0.05 and the means that exhibited significant differences were compared using Tukey’s significance test. All statistical analyses were performed using the SPSS Statistical Package (SPSS 17.0, Chicago, IL, USA).

The multiple linear regression equations of soil sucrase activity (Y1), protease activity (Y2), urease activity (Y3), acid phosphatase activity (Y4), catalase activity (Y5) and soil nutrient factors are as follows:

The multiple linear regression equations in PMM are:

Y1 = –0.534X1 + 0.821X2 + 0.173X3 − 0.258X4 − 0.012X5 − 0.065X6 − 0.257X7 (1)

Y2 = 0.689X1 + 0.354X2 − 0.392X3 + 0.020X4 + 0.225X5 + 0.524X6 − 0.195X7 (2)

Y3 = −0.021X1 + 0.443X2 − 0.394X3 − 0.157X4 − 0.115X5 + 0.233X6 + 0.275X7 (3)

Y4 = 1.045X1 − 0.128X2 − 0.042X3 + 0.080X4 + 0.134X5 + 0.069X6 + 0.048X7 (4)

Y5 = 2.023X1 − 2.069X2 − 0.181X3 + 0.065X4 + 0.065X5 + 0.244X6 + 0.169X7 (5)

The multiple linear regression equations in CPM are:

Y1 = 0.104X1 + 0.517X2 − 0.297X3 − 0.046X4 + 0.018X5 − 0.219X6 + 0.329X7 (6)

Y2 = 0.469X1 − 0.018X2 − 0.283X3 + 0.208X4 + 0.179X5 − 0.037X6 + 0.25X7 (7)

Y3 = 0.707X1 − 0.038X2 − 0.028X3 + 0.161X4 + 0.0009X5 − 0.017X6 + 0.196X7 (8)

Y4 = 0.219X1 + 0.239X2 + 0.053X3 − 0.139X4 + 0.154X5 − 0.131X6 + 0.031X7 (9)

Y5 = 0.243X1 + 0.143X2 + 0.197X3 − 0.037X4 + 0.015X5 − 0.274X6 + 0.264X7 (10)

The multiple linear regression equations in CMM are:

Y1 = 0.009X1 + 0.213X2 − 0.368X3 − 0.089X4 + 0.001X5 + 0.532X6 + 0.248X7 (11)

Y2 = 0.070X1 − 0.041X2 + 0.905X3 + 0.110X4 − 0.557X5 − 0.483X6 − 0.506X7 (12)

Y3 = 0.111X1 + 0.278X2 − 0.142X3 + 0.309X4 + 0.216X5 + 0.368X6 + 0.072X7 (13)

Y4 = 0.043X1 + 0.445X2 − 0.598X3 + 0.249X4 + 0.148X5 + 0.885X6 − 0.015X7 (14)

Y5 = −0.200X1 + 0.436X2 + 0.088X3 − 0.132X4 − 0.199X5 + 0.174X6 + 0.274X7 (15)

X1, X2, X3, X4, X5, X6 and X7 represent SOC, TN, TP, TK, AN, AP and AK, respectively. These equations present the direct path coefficients. The indirect path coefficients were obtained using the direct path coefficients multiplied by each soil nutrient’s correlation coefficient. The direct path coefficient was the direct effect of soil nutrient factors on enzyme activity.

## 5. Conclusions

In our study, the activity of enzymes, SOC, and TN was higher in the topsoil than the subsoil because of the higher accumulation of SOM on the topsoil and the much favorable moisture and temperature conditions of topsoil. SOC and most of the soil total nutrient content and nutrient availability were significantly higher in the intercropping system. Moreover, apart from soil catalase and urease, other enzymes’ soil enzymatic activities were higher in the intercropping than in the mono-cropping system. Path analysis provides the weighted effect of soil nutrients on soil enzymes in each cropping type. Intercropping may also lead to an increase in the other soil quality indicators, such as SOM. Intercropping of peanut with tea could significantly increase soil fertility. The long-term sustainability of the soil ecosystem in tea farming can be achieved with the help of peanut–tea intercropping.

## Figures and Tables

**Table 1:** Soil organic carbon (g.kg^-1^) content at different soil depths in *C. oleifera* mono-cropping model (CMM), peanut mono-cropping model (PMM), and Camellia–peanut inter-cropping model (CPM).

Cropping Model	0–10 cm	10–20 cm	20–30 cm	30–40 cm
PMM	19.93 ± 1.5 ^Aa^	16.61 ± 1.39 ^Ab^	8.62 ± 2.11 ^Ac^	6.56 ± 1.79 ^Ac^
CPM	16.77 ± 0.77 ^Ba^	12.56 ± 4.48 ^Ba^	14.37 ± 6.17 ^Bab^	7.68 ± 2.22 ^Bb^
CMM	13.9 ± 1.65 ^Ca^	12.15 ± 1.93 ^Ba^	9.89 ± 3.04 ^Ca^	5.61 ± 3.11 ^Cb^

**Note:** Two-way analysis of variance (ANOVA) test was performed and the values are mean ± SD. Different uppercase letters indicate significant differences between different cropping systems at *p* < 0.05, while different lowercase letters indicate significant differences in different depths *p* < 0.05.

**Table 2:** Correlation analysis of soil nutrients and enzyme activities in *C. oleifera* mono-cropping model (CMM), peanut mono-cropping model (PMM), and the Camellia–peanut inter-cropping model (CPM).

Cropping Model	Soil Enzyme	SOC	TN	TP	TK	AN	AP	AK
PMM	Sucrase	0.21	0.22	0.04	−0.27	0.01	0.08	0.07
	Protease	0.45	0.47	−0.05	−0.14	0.29	0.37	0.17
	Urease	0.68 *	0.71 *	−0.15	−0.5 *	−0.14	0.25	0.58 *
	Acid phosphatase	0.94 *	0.92 **	0.13	−0.29	0.19	0.41	0.57 *
	Catalase	0.17	0.05	0.06	−0.02	−0.11	0.2	−0.03
CPM	Sucrase	0.33	0.51 *	−0.08	0	0.11	−0.18	0.22
	Protease	0.56 *	0.21	0.11	0.2	0.39	0.32	0.38
	Urease	0.75 *	0.26	0.32	0.23	0.43 *	0.03	0.46 *
	Acid phosphatase	0.42 **	0.34	0.15	−0.11	0.33	−0.03	0.13
	Catalase	0.44 *	0.26	0.34	0.12	0.25	−0.14	0.41 *
CMM	Sucrase	0.35	0.41	0.38	−0.05	0.03	0.41 *	0.31
	Protease	0.05	−0.01	0.17	−0.07	−0.55 *	0.19	−0.31
	Urease	0.41 *	0.52 *	0.52 *	0.23	0.23	0.48 *	0.49 *
	Acid phosphatase	0.51 **	0.62 *	0.58 *	0.11	0.12	0.62 *	0.44 **
	Catalase	0.43 *	0.52 *	0.54 *	0.01	−0.08	0.54 *	0.42 *

**Note:** Significant values are bold. Values given are the Pearson correlation coefficients. * Correlation is significant at the 0.05 level (2-tailed). ** Correlation is significant at the 0.01 level (2-tailed).

**Table 3:** Two-way ANOVA results for soil variables. *, ** and *** indicate a significant level at *p* < 0.05, *p* < 0.01 and *p* < 0.001, respectively.

	Cropping Model		Soil Depth		Cropping Model × Soil Depth	
Variable	*F*	*P*	*F*	*P*	*F*	*P*
TN	122.6	**	1233.6	*	365.5	**
TP	870.2	*	1364.4	**	102.1	***
TK	683.3	**	65.3	0.274	89.2	**
AN	42.9	**	78.3	**	51.2	**
AP	632.4	**	896.7	**	3546.5	**
AK	536.2	*	336.3	***	1456.5	**
Protease	256.3	0.066	256.5	**	785.4	0.064
Sucrase	158.9	**	447.3	**	1235.9	*
A. phosphatase	1244.3	*	380.3	0.066	3225.0	**
Urease	1563.2	**	125.6	**	2132.5	**
Catalase	853.2	**	0.072	**	125.2	**

## Data Availability

No additional data available.

## Appendix A

**Table A1:** Path coefficients between soil nutrients factors (X) and soil enzyme activity (Y) in PMM.

Factors Code		X1	X2	X3	X4	X5	X6	X7
Y1	X1	−0.534 *	−0.518	−0.048	0.219	−0.021	−0.198	−0.315
	X2	0.796	0.821 *	0.066	−0.320	0.099	0.304	0.550
	X3	0.016	0.014	0.173 *	0.014	0.022	0.112	0.054
	X4	0.106	0.101	−0.021	−0.258 *	−0.080	0.041	0.065
	X5	0.000	−0.001	−0.002	−0.004	−0.012 *	−0.001	0.000
	X6	−0.024	−0.024	−0.042	0.010	−0.007	−0.065 *	−0.027
	X7	−0.152	−0.172	−0.080	0.064	0.005	−0.105	−0.257 *
Y2	X1	0.689 *	0.668	0.062	−0.282	0.028	0.255	0.407
	X2	0.343	0.354 *	0.028	−0.138	0.042	0.131	0.237
	X3	−0.035	−0.031	−0.392 *	−0.031	−0.051	−0.255	−0.122
	X4	−0.008	−0.008	0.002	0.020*	0.006	−0.003	−0.005
	X5	0.009	0.027	0.029	0.070	0.225 *	0.025	−0.005
	X6	0.194	0.194	0.341	−0.084	0.058	0.524 *	0.215
	X7	−0.115	−0.131	−0.060	0.049	0.004	−0.080	−0.195 *
Y3	X1	−0.021 *	−0.020	−0.002	0.009	−0.001	−0.008	−0.012
	X2	0.430	0.443 *	0.035	−0.173	0.053	0.164	0.297
	X3	−0.035	−0.032	−0.394 *	−0.032	−0.051	−0.256	−0.122
	X4	0.064	0.061	−0.013	−0.157 *	−0.049	0.025	0.039
	X5	−0.005	−0.014	−0.015	−0.036	−0.115 *	−0.013	0.002
	X6	0.086	0.086	0.151	−0.037	0.026	0.233 *	0.096
	X7	0.162	0.184	0.085	−0.069	−0.006	0.113	0.275 *
Y4	X1	1.045 *	1.014	0.094	−0.428	0.042	0.387	0.617
	X2	−0.124	−0.128 *	−0.010	0.050	−0.015	−0.047	−0.086
	X3	−0.004	−0.003	−0.042 *	−0.003	−0.005	−0.027	−0.013
	X4	−0.033	−0.031	0.006	0.080 *	0.025	−0.013	−0.020
	X5	0.005	0.016	0.017	0.042	0.134 *	0.015	−0.003
	X6	0.026	0.026	0.045	−0.011	0.008	0.069 *	0.028
	X7	0.028	0.032	0.015	−0.012	−0.001	0.020	0.048 *
Y5	X1	2.023 *	1.962	0.182	−0.829	0.081	0.749	1.194
	X2	−2.007	−0.069 *	−0.166	0.807	−0.248	−0.766	−1.386
	X3	−0.016	−0.014	−0.181 *	−0.014	−0.024	−0.118	−0.056
	X4	−0.027	−0.025	0.005	0.065 *	0.020	−0.010	−0.016
	X5	0.003	0.008	0.008	0.020	0.065 *	0.007	−0.001
	X6	0.090	0.090	0.159	−0.039	0.027	0.244 *	0.100
	X7	0.100	0.113	0.052	−0.042	−0.003	0.069	0.169 *

* indicate a significant level at *p* < 0.05.

**Table A2:** Path coefficients between soil nutrients factors (X) and soil enzyme activity (Y) in in CPM.

Factors Code		X1	X2	X3	X4	X5	X6	X7
Y1	X1	0.104 *	0.202	−0.086	0.000	0.010	−0.007	0.105
	X2	0.041	0.517 *	−0.050	−0.003	0.004	−0.033	0.039
	X3	0.030	0.088	−0.297 *	−0.015	0.007	−0.068	0.174
	X4	0.000	0.036	−0.098	−0.046 *	−0.001	−0.018	0.132
	X5	0.059	0.114	−0.113	0.002	0.018 *	−0.072	0.102
	X6	0.003	0.078	−0.092	−0.004	0.006	−0.219 *	0.049
	X7	0.033	0.062	−0.157	−0.018	0.006	−0.033	0.329 *
Y2	X1	0.469 *	−0.007	−0.082	0.000	0.102	−0.001	0.080
	X2	0.183	−0.018 *	−0.048	0.015	0.039	−0.006	0.030
	X3	0.136	−0.003	−0.283 *	0.069	0.068	−0.011	0.133
	X4	0.000	−0.001	−0.093	0.208 *	−0.009	−0.003	0.100
	X5	0.267	−0.004	−0.108	−0.010	0.179 *	−0.012	0.078
	X6	0.014	−0.003	−0.088	0.017	0.059	−0.037 *	0.038
	X7	0.150	−0.002	−0.150	0.083	0.055	−0.006	0.250 *
Y3	X1	0.707 *	−0.015	−0.008	0.000	0.001	−0.001	0.063
	X2	0.276	−0.038 *	−0.005	0.011	0.000	−0.003	0.024
	X3	0.205	−0.006	−0.028 *	0.053	0.000	−0.005	0.104
	X4	0.000	−0.003	−0.009	0.161 *	0.000	−0.001	0.078
	X5	0.403	−0.008	−0.011	−0.008	0.001 *	−0.006	0.061
	X6	0.021	−0.006	−0.009	0.013	0.000	−0.017 *	0.029
	X7	0.226	−0.005	−0.015	0.064	0.000	−0.003	0.196 *
Y4	X1	0.219 *	0.093	0.015	0.000	0.088	−0.004	0.010
	X2	0.085	0.239 *	0.009	−0.010	0.034	−0.020	0.004
	X3	0.064	0.041	0.053 *	−0.046	0.059	−0.041	0.016
	X4	0.000	0.017	0.017	−0.139 *	−0.008	−0.010	0.012
	X5	0.125	0.053	0.020	0.007	0.154 *	−0.043	0.010
	X6	0.007	0.036	0.016	−0.011	0.051	−0.131 *	0.005
	X7	0.070	0.029	0.028	−0.056	0.048	−0.020	0.031 *
Y5	X1	0.243 *	0.056	0.057	0.000	0.009	−0.008	0.084
	X2	0.095	0.143 *	0.033	−0.003	0.003	−0.041	0.032
	X3	0.070	0.024	0.197 *	−0.012	0.006	−0.085	0.140
	X4	0.000	0.010	0.065	−0.037 *	−0.001	−0.022	0.106
	X5	0.139	0.031	0.075	0.002	0.015 *	−0.090	0.082
	X6	0.007	0.021	0.061	−0.003	0.005	−0.274 *	0.040
	X7	0.078	0.017	0.104	−0.015	0.005	−0.041	0.264 *

* indicate a significant level at *p* < 0.05.

**Table A3:** Path coefficients between soil nutrients factors (X) and soil enzyme activity (Y) in in CMM.

Factors Code		X1	X2	X3	X4	X5	X6	X7
Y1	X1	0.009 *	0.083	−0.107	0.000	0.001	0.016	0.079
	X2	0.004	0.213 *	−0.063	−0.006	0.000	0.080	0.037
	X3	0.003	0.036	−0.368 *	−0.029	0.000	0.165	0.131
	X4	0.000	0.015	−0.121	−0.089 *	0.000	0.043	0.099
	X5	0.005	0.047	−0.140	0.004	0.001 *	0.176	0.077
	X6	0.000	0.032	−0.114	−0.007	0.000	0.532 *	0.037
	X7	0.003	0.026	−0.195	−0.036	0.000	0.080	0.248 *
Y2	X1	0.070 *	−0.016	0.262	0.000	−0.317	−0.014	−0.162
	X2	0.027	−0.041 *	0.154	0.008	−0.123	−0.072	−0.076
	X3	0.020	−0.007	0.905 *	0.036	−0.212	−0.150	−0.268
	X4	0.000	−0.003	0.299	0.110 *	0.028	−0.039	−0.202
	X5	0.040	−0.009	0.344	−0.006	−0.557 *	−0.159	−0.157
	X6	0.002	−0.006	0.281	0.009	−0.184	−0.483 *	−0.076
	X7	0.022	−0.005	0.480	0.044	−0.173	−0.072	−0.506 *
Y3	X1	0.111 *	0.108	−0.041	0.000	0.123	0.011	0.023
	X2	0.043	0.278 *	−0.024	0.022	0.048	0.055	0.011
	X3	0.032	0.047	−0.142 *	0.102	0.082	0.114	0.038
	X4	0.000	0.019	−0.047	0.309 *	−0.011	0.029	0.029
	X5	0.063	0.061	−0.054	−0.015	0.216 *	0.121	0.022
	X6	0.003	0.042	−0.044	0.025	0.071	0.368 *	0.011
	X7	0.036	0.033	−0.075	0.124	0.067	0.055	0.072 *
Y4	X1	0.043 *	0.174	−0.173	0.000	0.084	0.027	−0.005
	X2	0.017	0.445 *	−0.102	0.017	0.033	0.133	−0.002
	X3	0.012	0.076	−0.598 *	0.082	0.056	0.274	−0.008
	X4	0.000	0.031	−0.197	0.249 *	−0.007	0.071	−0.006
	X5	0.025	0.098	−0.227	−0.012	0.148 *	0.292	−0.005
	X6	0.001	0.067	−0.185	0.020	0.049	0.885 *	−0.002
	X7	0.014	0.053	−0.317	0.100	0.046	0.133	−0.015 *
Y5	X1	−0.200 *	0.170	0.026	0.000	−0.113	0.005	0.088
	X2	−0.078	0.436 *	0.015	−0.009	−0.044	0.026	0.041
	X3	−0.058	0.074	0.088 *	−0.044	−0.076	0.054	0.145
	X4	0.000	0.031	0.029	−0.132 *	0.010	0.014	0.110
	X5	−0.114	0.096	0.033	0.007	−0.199 *	0.057	0.085
	X6	−0.006	0.065	0.027	−0.011	−0.066	0.174 *	0.041
	X7	−0.064	0.052	0.047	−0.053	−0.062	0.026	0.274 *

* indicate a significant level at *p* < 0.05.

## References

[1] Brooker R.W., Bennett A.E., Cong W., Daniell T.J., George T.S., Hallett P.D., Hawes C., Iannetta P.P.M., Jones H.G., Karley A.J. (2015). Improving intercropping: A synthesis of research in agronomy, plant physiology and ecology. New Phytol..

[2] Ananthi T., Amanullah M.M., Al-Tawaha A.R.M.S. (2017). A review on maize-legume intercropping for enhancing the productivity and soil fertility for sustainable agriculture in India. Adv. Environ. Biol..

[3] Farooq T.H., Shakoor A., Wu X., Li Y., Rashid M.H.U., Zhang X., Gilani M.M., Kumar U., Chen X., Yan W. (2021). Perspectives of plantation forests in the sustainable forest development of China. iForest.

[4] Farooq T.H., Yan W., Rashid M.H.U., Tigabu M., Gilani M.M., Zou X.H., Wu P.F. (2019). Chinese fir (*Cunninghamia Lanceolata*) a green gold of China with continues decline in its productivity over the successive rotations: A review. Appl. Ecol. Environ. Res..

[5] Farooq T.H., Wu W., Tigabu M., Ma X., He Z., Rashid M.H., Gilani M.M., Wu P.F. (2019). Growth, biomass production and root development of Chinese fir in relation to initial planting density. Appl. Ecol. Environ. Res..

[6] Farooq T.H., Ma X., Rashid M.H.U., Wu W., Xu J., Tarin M.W.K., He Z., Wu P. (2019). Impact of stand density on soil quality in Chinese Fir (*Cunninghamia Lanceolata*) monoculture. Appl. Ecol. Environ. Res..

[7] Powlson D.S., Gregory P.J., Whalley W.R., Quinton J.N., Hopkins D.W., Whitmore A.P., Hirsch P.R., Goulding K.W.T. (2011). Soil management in relation to sustainable agriculture and ecosystem services. Food Policy.

[8] Ghosh P.K., Bandyopadhyay K.K., Wanjari R.H., Manna M.C., Misra A.K., Mohanty M., Rao A.S. (2007). Legume effect for enhancing productivity and nutrient use-efficiency in major cropping systems–an Indian perspective: A review. J. Sustain. Agric..

[9] Rowe H., Withers P.J.A., Baas P., Chan N.I., Doody D., Holiman J., Jacobs B., Li H., MacDonald G.K., McDowell R. (2016). Integrating legacy soil phosphorus into sustainable nutrient management strategies for future food, bioenergy and water security. Nutr. Cycl. Agro Ecosyst..

[10] Gebru H. (2015). A review on the comparative advantages of intercropping to mono-cropping system. J. Biol. Agric. Healthc..

[11] Powell J.M., Williams T.O. (1993). Livestock, Nutrient Cycling and Sustainable Agriculture in the West African Sahel.

[12] Nyawade S.O., Gachene C.K.K., Karanja N.N., Gitari H.I., Schulte-Geldermann E., Parker M.L. (2019). Controlling soil erosion in smallholder potato farming systems using legume intercrops. Geoderma Reg..

[13] Seran T.H., Brintha I. (2010). Review on maize based intercropping. J. Agron..

[14] Dong B., Wu B., Hong W., Li X., Li Z., Xue L., Huang Y. (2017). Transcriptome analysis of the tea oil camellia (*Camellia oleifera*) reveals candidate drought stress genes. PLoS ONE.

[15] Akram N.A., Shafiq F., Ashraf M. (2018). Peanut (*Arachis hypogaea* L.): A prospective legume crop to offer multiple health benefits under changing climate. Compr. Rev. Food Sci. Food Saf..

[16] Li Q., Wu L., Jun C., Khan M.A., Luo X., Lin W. (2016). Biochemical and microbial properties of rhizospheres under maize/peanut intercropping. J. Integr. Agric..

[17] He Y., Ding N., Shi J., Wu M., Liao H., Xu J. (2013). Profiling of microbial PLFAs: Implications for interspecific interactions due to intercropping which increase phosphorus uptake in phosphorus limited acidic soils. Soil Biol. Biochem..

[18] Burns R.G., DeForest J.L., Marxsen J., Sinsabaugh R.L., Stromberger M.E., Wallenstein M.D., Weintraub M.N., Zoppini A. (2013). Soil enzymes in a changing environment: Current knowledge and future directions. Soil Biol. Biochem..

[19] Das S.K., Varma A. (2010). Role of enzymes in maintaining soil health. Soil Enzymology.

[20] Neher D.A. (2001). Role of nematodes in soil health and their use as indicators. J. Nematol..

[21] Farooq T.H., Tigabu M., Ma X., Zou X., Liu A., Odén P.C., Wu P. (2018). Nutrient uptake, allocation and biochemical changes in two Chinese fir cuttings under heterogeneous phosphorus supply. IForest.

[22] Farooq T.H., Yan W., Chen X., Shakoor A., Rashid M.H.U., Gilani M.M., He Z., Wu P. (2020). Dynamics of canopy development of *Cunninghamia lanceolata* mid-age plantation in relation to foliar nitrogen and soil quality influenced by stand density. Glob. Ecol. Conserv..

[23] Wu P., Wang G., Farooq T.H., Li Q., Zou X., Ma X. (2017). Low phosphorus and competition affect Chinese fir cutting growth and root organic acid content: Does neighboring root activity aggravate P nutrient deficiency?. J. Soils Sediments.

[24] Loveland P., Webb J. (2003). Is there a critical level of organic matter in the agricultural soils of temperate regions: A review. Soil Tillage Res..

[25] Jiang F., Drohan P.J., Cibin R., Preisendanz H.E., White C.M., Veith T.L. (2021). Reallocating crop rotation patterns improves water quality and maintains crop yield. Agric. Syst..

[26] Li W., Zheng Z., Li T., Zhang X., Wang Y., Yu H., He S., Liu T. (2015). Effect of tea plantation age on the distribution of soil organic carbon fractions within water-stable aggregates in the hilly region of Western Sichuan, China. Catena.

[27] Lu W., Shen X., Chen Y. (2019). Effects of intercropping peanut on soil nutrient status and microbial activity within young Camellia oleifera plantation. Commun. Soil Sci. Plant Anal..

[28] Hu C.H., Wang P.Q., Zhang P.P., Nie X.M., Li B.B., Tai L., Liu W.T., Li W.Q., Chen K.M. (2020). NADPH oxidases: The vital performers and center hubs during plant growth and signaling. Cells.

[29] Cameron K.C., Di H.J., Moir J.L. (2013). Nitrogen losses from the soil/plant system: A review. Ann. Appl. Biol..

[30] Dubey R.S., Pessarakli M. (1995). Physiological mechanisms of nitrogen absorption and assimilation in plants under stressful conditions. Handb. Plant Crop Physiol..

[31] Jensen E.S. (1996). Grain yield, symbiotic N_2_ fixation and interspecific competition for inorganic N in pea-barley intercrops. Plant Soil.

[32] Jensen E.S., Carlsson G., Hauggaard-Nielsen H. (2020). Intercropping of grain legumes and cereals improves the use of soil N resources and reduces the requirement for synthetic fertilizer N: A global-scale analysis. Agron. Sustain. Dev..

[33] Xu B.C., Li F.M., Shan L. (2008). Switchgrass and milkvetch intercropping under 2: 1 row-replacement in semiarid region, northwest China: Aboveground biomass and water use efficiency. Eur. J. Agron..

[34] Ikramul Haq M., Maqbool M.M., Ali A., Farooq S., Khan S., Saddiq M.S., Khan K.A., Ali S., Ifnan Khan M., Hussain A. (2020). Optimizing planting geometry for Barley-Egyptian clover intercropping system in semi-arid sub-tropical climate. PLoS ONE.

[35] Giller K.E. (2001). Nitrogen Fixation in Tropical Cropping Systems.

[36] Singh D.K., Kumar S. (2008). Nitrate reductase, arginine deaminase, urease and dehydrogenase activities in natural soil (ridges with forest) and in cotton soil after acetamiprid treatments. Chemosphere.

[37] Yu S., Xue L., Feng Y., Liu Y., Song Z., Mandal S., Yang L., Sun Q., Xing B. (2020). Hydrochar reduced NH_3_ volatilization from rice paddy soil: Microbial-aging rather than water-washing is recommended before application. J. Clean. Prod..

[38] Li L., Li S.-M., Sun J.H., Zhou L.L., Bao X.G., Zhang H.-G., Zhang F.-S. (2007). Diversity enhances agricultural productivity via rhizosphere phosphorus facilitation on phosphorus-deficient soils. Proc. Natl. Acad. Sci. USA.

[39] Shen J., Li C., Mi G., Li L., Yuan L., Jiang R., Zhang F. (2013). Maximizing root/rhizosphere efficiency to improve crop productivity and nutrient use efficiency in intensive agriculture of China. J. Exp. Bot..

[40] Wang X., Gao Y., Zhang H., Shao Z., Sun B., Gao Q. (2020). Enhancement of rhizosphere citric acid and decrease of NO_3_−/NH_4_+ ratio by root interactions facilitate N fixation and transfer. Plant Soil.

[41] Rasool B., Ramzani P.M.A., Zubair M., Khan M.A., Lewińska K., Turan V., Karczewska A., Khan S.A., Farhad M., Tauqeer H.M. (2021). Impacts of oxalic acid-activated phosphate rock and root-induced changes on lead bioavailability in rhizosphere and distribution in mung bean plant. Environ. Pollut..

[42] Maurya P.R., Lal R. (1981). Effects of different mulch materials on soil properties and on the root growth and yield of maize (*Zea mays*) and cowpea (*Vigna unguiculata*). F. Crop. Res..

[43] Dick R.P. (1994). Soil enzyme activities as indicators of soil quality. Defin. Soil Qual. Sustain. Environ..

[44] Sherene T. (2017). Role of soil enzymes in nutrient transformation: A review. Bio. Bull..

[45] Han S., Delgado-Baquerizo M., Luo X., Liu Y., Van Nostrand J.D., Chen W., Zhou J., Huang Q. (2021). Soil aggregate size-dependent relationships between microbial functional diversity and multifunctionality. Soil Biol. Biochem..

[46] Roohi M., Arif M.S., Yasmeen T., Riaz M., Rizwan M., Shahzad S.M., Ali S., Bragazza L. (2020). Effects of cropping system and fertilization regime on soil phosphorous are mediated by rhizosphere-microbial processes in a semi-arid agroecosystem. J. Environ. Manag..

[47] Jan M.T., Roberts P., Tonheim S.K., Jones D.L. (2009). Protein breakdown represents a major bottleneck in nitrogen cycling in grassland soils. Soil Biol. Biochem..

[48] Kwiatkowski C.A., Harasim E., Feledyn-Szewczyk B., Antonkiewicz J. (2020). Enzymatic activity of loess soil in organic and conventional farming systems. Agriculture.

[49] Chen L., Rossi F., Deng S., Liu Y., Wang G., Adessi A., De Philippis R. (2014). Macromolecular and chemical features of the excreted extracellular polysaccharides in induced biological soil crusts of different ages. Soil Biol. Biochem..

[50] Wang Y., Dong J., Zheng X., Zhang J., Zhou P., Song X., Song W., Wang S. (2021). Wheat straw and biochar effect on soil carbon fractions, enzyme activities, and nutrients in a tobacco field. Can. J. Soil Sci..

[51] Rashid M.H.U., Tigabu M., Chen H., Farooq T.H., Ma X., Wu P. (2020). Calcium-mediated adaptive responses to low phosphorus stress in Chinese fir. Trees.

[52] Deng S.P., Tabatabai M.A. (1997). Effect of tillage and residue management on enzyme activities in soils: III. Phosphatases and arylsulfatase. Biol. Fertil. Soils.

[53] Zhang W., Gruszewski H.A., Chevone B.I., Nessler C.L. (2008). An Arabidopsis purple acid phosphatase with phytase activity increases foliar ascorbate. Plant Physiol..

[54] Uren N.C. (2007). Types, amounts, and possible functions of compounds released into the rhizosphere by soil-grown plants. Rhizosph. Biochem. Org. Subst. Soil Plant Interface.

[55] Wen F., VanEtten H.D., Tsaprailis G., Hawes M.C. (2007). Extracellular proteins in pea root tip and border cell exudates. Plant Physiol..

[56] Mobley H.L.T., Hu L.-T., Foxall P.A. (1991). Helicobacter pylori urease: Properties and role in pathogenesis. Scand. J. Gastroenterol..

[57] Ansari M.W., Trivedi D.K., Sahoo R.K., Gill S.S., Tuteja N. (2013). A critical review on fungi mediated plant responses with special emphasis to Piriformospora indica on improved production and protection of crops. Plant Physiol. Biochem..

[58] Li Z.H., Wang Q., Ruan X., Pan C.D., Jiang D.A. (2010). Phenolics and plant allelopathy. Molecules.

[59] Tian Y., Cao F., Wang G. (2013). Soil microbiological properties and enzyme activity in Ginkgo–tea agroforestry compared with monoculture. Agrofor. Syst..

[60] Udawatta R.P., Kremer R.J., Adamson B.W., Anderson S.H. (2008). Variations in soil aggregate stability and enzyme activities in a temperate agroforestry practice. Appl. Soil Ecol..

[61] Shakoor A., Shakoor S., Rehman A., Ashraf F., Abdullah M., Shahzad S.M., Farooq T.H., Ashraf M., Manzoor M.A., Altaf M. (2020). Effect of animal manure, crop type, climate zone, and soil attributes on greenhouse gas emissions from agricultural soils—A global meta-analysis. J. Clean. Prod..

[62] Shakoor A., Shahzad S.M., Chatterjee N., Arif M.S., Farooq T.H., Altaf M.M., Tufail M.A., Dar A.A., Mehmood T. (2021). Nitrous oxide emission from agricultural soils: Application of animal manure or biochar? A global meta-analysis. J. Environ. Manag..

[63] Shakoor A., Shahbaz M., Farooq T.H., Sahar N.E., Shahzad S.M., Altaf M.M., Ashraf M. (2021). A global meta-analysis of greenhouse gases emission and crop yield under no-tillage as compared to conventional tillage. Sci. Total Environ..

[64] Hu W., Jiao Z., Wu F., Liu Y., Dong M., Ma X., Fan T., An L., Feng H. (2014). Long-term effects of fertilizer on soil enzymatic activity of wheat field soil in Loess Plateau, China. Ecotoxicology.

[65] Yang L., Li T., Li F., Lemcoff J.H., Cohen S. (2008). Fertilization regulates soil enzymatic activity and fertility dynamics in a cucumber field. Sci. Hortic..

[66] Zhao S., Chen X., Deng S., Dong X., Song A., Yao J., Fang W., Chen F. (2016). The effects of fungicide, soil fumigant, bio-organic fertilizer and their combined application on *chrysanthemum Fusarium* wilt controlling, soil enzyme activities and microbial properties. Molecules.

[67] Lu N., Yu M., Cui M., Luo Z., Feng Y., Cao S., Sun Y., Li Y. (2016). Effects of different ectomycorrhizal fungal inoculates on the growth of *Pinus tabulaeformis* seedlings under greenhouse conditions. Forests.

[68] Ryan J., Estefan G., Rashid A. (2001). Soil and Plant Analysis Laboratory Manual.

[69] Bray R.H., Kurtz L.T. (1945). Determination of total, organic, and available forms of phosphorus in soils. Soil Sci..

[70] Schollenberger C.J., Simon R.H. (1945). Determination of exchange capacity and exchangeable bases in soil—ammonium acetate method. Soil Sci..

